# Case Report: Effectiveness of Targeted Treatment in a Patient With Pancreatic Cancer Harboring PALB2 Germline Mutation and KRAS Somatic Mutation

**DOI:** 10.3389/fmed.2021.746637

**Published:** 2022-01-13

**Authors:** Wei Wu, Yu Liu, Yuzhi Jin, Lulu Liu, Yixuan Guo, Mian Xu, Qing Hao, Dazhi Li, Weijia Fang, Aibin Zhang, Peng Zhao

**Affiliations:** ^1^Department of Medical Oncology, The First Affiliated Hospital, College of Medicine, Zhejiang University & Key Laboratory of Cancer Prevention and Intervention, Ministry of Education, Hangzhou, Zhejiang, China; ^2^OrigiMed, Shanghai, China; ^3^Division of Hepatobiliary and Pancreatic Surgery, Department of Surgery, The First Affiliated Hospital, College of Medicine, Zhejiang University, Hangzhou, China

**Keywords:** pancreatic cancer, PALB2 germline mutation, KRAS somatic mutation, targeted therapy, case report

## Abstract

Pancreatic cancer is one of the most leading causes of cancer death worldwide. The rapid development of next-generation sequencing (NGS) and precision medicine promote us to seek potential targets for the treatment of pancreatic cancer. Here, we report a female pancreatic cancer patient who underwent radical surgical excision after neoadjuvant chemotherapy. After the surgery, the patient underwent gemcitabine + S-1 therapy, capecitabine + albumin paclitaxel therapy and irinotecan therapy successively, however, MRI review revealed tumor progression. The surgical tissue sample was subjected to next-generation sequencing (NGS), and PALB2 germline mutation and KRAS somatic mutation were identified. The patient then received olaparib (a PARP inhibitor) + irinotecan and the disease stabilized for one year. Due to the increased CA19-9, treatment of the patient with a combination of trametinib (a MEK inhibitor) and hydroxychloroquine resulted in stable disease (SD) with a significant decrease of CA19-9. This case demonstrated that the NGS may be a reliable method for finding potential therapeutic targets for pancreatic cancer.

## Introduction

Pancreatic cancer is one of the most deadly malignant tumors in the world (1). So far, surgical resection is still the primary treatment strategy for pancreatic cancer. However, most patients lose the opportunity to undergo radical surgery due to late-stage diagnosis (2). The clinical outcomes of pancreatic cancer remain poor due to limited effective treatments and a high rate of metastasis and recurrence (3). Genetic and epigenetic aberrations related to abnormal activation of tumor driver genes are often found in pancreatic cancer (4). Therefore, targeted therapy may be an important approach to improve the overall survival of pancreatic cancer patients.

Partner and localizer BRCA2 (PALB2) is an important DNA repair gene and plays an essential role in homologous recombination. It links BRCA1 and BRCA2 (5), enabling recombination repair and checkpoint functions in maintaining genomic integrity (6). In addition, it also participates in double-stranded break repair during DNA damage response (7). PALB2 gene mutation mainly includes non-sense mutations, frameshift mutations and loss-of-function mutations (8), and PALB2 is identified as a susceptibility gene of pancreatic cancer (9), familial breast cancer (10) and ovarian cancer (11).

The rat sarcoma virus (RAS) family includes more than 150 small monomeric proteins with GTPase activity. Kirsten rat sarcoma viral oncogene homolog (KRAS) belongs to the RAS family, and mutations in KRAS will affect RAS-mediated GTP hydrolysis, leading to the continuous activation of RAS and thereby promoting the malignant transformation of cells (12). KRAS gene activation mutations and amplification are very common in a variety of malignant diseases, such as colon cancer, lung cancer, thyroid cancer and pancreatic cancer (13). It is generally considered that KRAS gene mutation is a poor prognostic factor for a variety of cancers, including pancreatic cancer (14). The cBioPortal database (2020) shows that the frequency of KRAS gene mutations in pancreatic adenocarcinoma is about 85.9–94.9%.

Given the limited data on targeted medication for PALB2 germline mutations and KRAS gene mutation in pancreatic cancer, we herein report a case of a pancreatic cancer patient with PALB2 germline mutation and KRAS mutation, and this patient responses well to targeted therapy.

## Case Presentation

The patient, female, 56 years old, was in good health and had no history of any chronic diseases. The patient attended our hospital in October 2016 due to pain in the left lower back and pain and discomfort under the xiphoid. Computed tomography (CT) scan showed space-occupying of the pancreatic tail, invading the abdominal aorta. Endoscopic ultrasonography-guided biopsy of the pancreatic tail was performed and the pathology revealed adenocarcinoma. After multiple disciplinary team (MDT) discussion, the patient was diagnosed with locally advanced pancreatic cancer, and neoadjuvant therapy is recommended first. From October 2016 to July 2017, the patient received GS chemotherapy (a total of 10 courses): Gemcitabine 1600 mg D1, D8 + S-1 60 mg Bid D1-14, Q3W. A restaging CT scan in January 2017 indicated the unavailability of radical resection. Hence, during GS chemotherapy, 25 rounds of radiotherapy started in January 2017. Magnetic resonance (MR) in July 2017 showed a mass in the pancreatic body with enlarged lymph nodes around the pancreas and retroperitoneum, accompanied by splenic arteriovenous invasion and multiple collateral circulations. Efficacy evaluation was partial response (PR) at that time. Pancreatectomy and splenectomy were performed in our hospital on July 25, 2017, and the pathology of the surgical tissue showed moderate-poorly differentiated adenocarcinoma. A surgical tissue sample was obtained for next-generation sequencing (NGS) using a panel of 450 cancer-related genes (OrigiMed, Shanghai, China) on July 25, 2017. Ultimately, PALB2 (D1168Efs^*^22) germline mutation and KRAS (G12R) somatic mutation were detected.

After the surgery, from September 2017 to November 2017, the patient was continued to receive GS chemotherapy and radiotherapy. However, on November 17, 2017, restaging MR displayed two abnormal lesions in the left liver, which were considered as liver metastases. Radiofrequency ablation was manipulated and capecitabine 1250 mg bid D1-14 + albumin paclitaxel 200 mg D1, D8, Q3W were given for 6 cycles as chemotherapy from December 2017 to May 2018. Nevertheless, the level of CA19-9 increased dramatically from August 2018 to September 2018, and restaging MR showed anterior abdominal wall mass and retroperitoneal mass, indicating tumor metastasis. Efficacy evaluation was progressive disease (PD).

**Figure 1:**
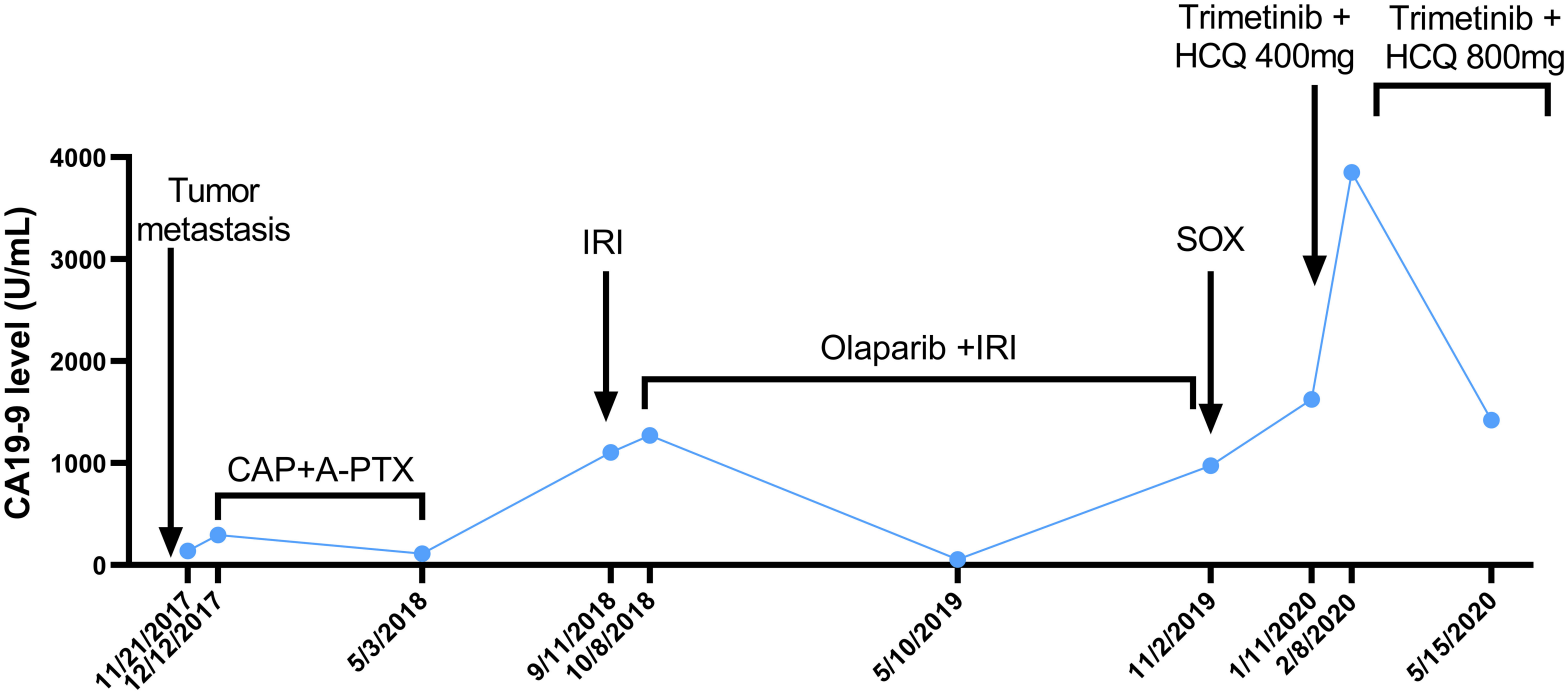
The curve of CA199 change after tumor metastasis. The patient's CA19-9 level was measured periodically and is annotated with the dates and treatments administered. A-PTX, albumin paclitaxel; CA19-9, carbohydrate antigen 19-9; CAP, capecitabine; HCQ, hydroxychloroquine; IRI, irinotecan; SOX, S-1 plus oxaliplatin.

**Figure 2:**
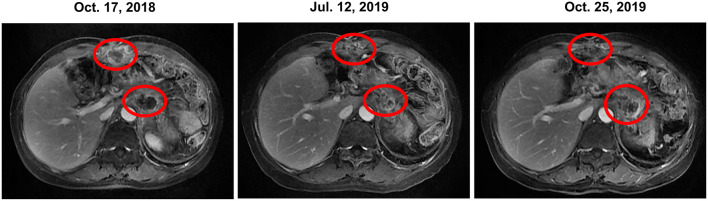
Change in the tumor prior to, during and following olaparib therapy based on representative MRI. In October 2018, prior to olaparib therapy, MRI showed anterior abdominal wall mass and retroperitoneal mass. Continuous stable disease was observed for 12 months during olaparib (with or without irinotecan) therapy. However, the retroperitoneal mass enlarged in October 2019, meanwhile, the CA19-9 level increased dramatically. Thus, the patient was transferred to SOX chemotherapy. CA19-9, carbohydrate antigen 19-9; MRI, magnetic resonance images; SOX, S-1 plus oxaliplatin.

The patient received irinotecan 250 mg Q3W as chemotherapy for 6 cycles from September 2018 to January 2019. During this period, due to the increase of CA19-9 level (Figure 1), Olaparib combined with irinotecan was used for treatment from October 2018, and the disease stabilized until November 2019 (Figure 2). Due to the increase of CA19-9 (Figure 1), the patient transferred to SOX chemotherapy (oxaliplatin injection 130 mg D1 + S-1 60 mg twice a day, D1-7, Q2W) for 5 cycles from 2019-11 to 2020-1. However, the volume of the anterior abdominal wall mass and retroperitoneal mass increased, indicating disease progression.

**Figure 3:**
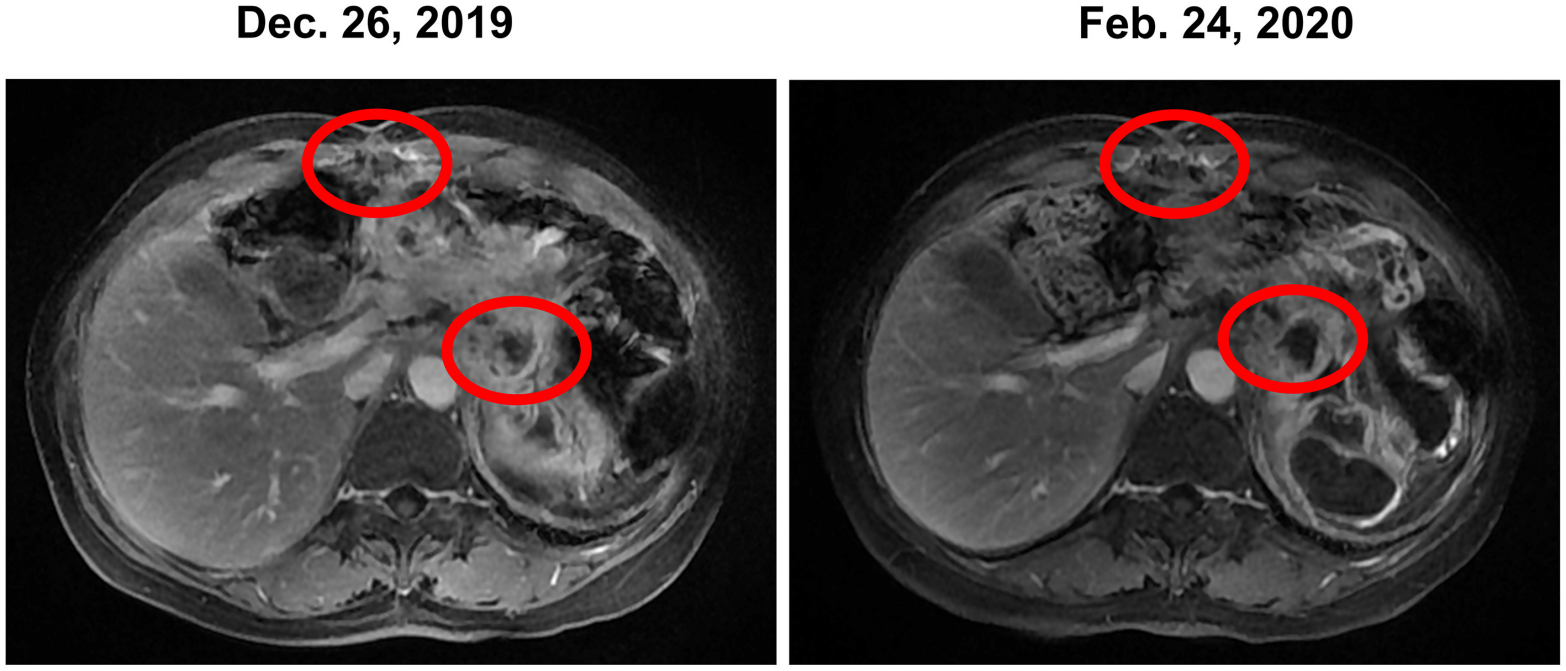
Stable disease was observed during the treatment of trametinib combined with hydroxychloroquine based on MRI. MRI, magnetic resonance images.

In December 2019, the patient began receiving trametinib (2 mg, daily) + hydroxychloroquine (200 mg, twice a day). Nevertheless, the patient's level of CA 19-9 (Figure 1) continued to increase during the first month of treatment. The hydroxychloroquine was escalated to 800 mg daily (400 mg, twice a day) and the trametinib dose was unchanged. During the three months of the treatment, she experienced grade 2 neutropenia and grade 1 fatigue. The CA19-9 level (Figure 1) decreased significantly, and MRI (Figure 3) indicated a 6% reduction in tumor burden by RECIST 1.1 criteria, achieving stable disease (SD).

For the further guidance of next treatment, the blood sample was used for NGS in May 2020, and the results still showed PALB2 (D1168Efs^*^22) germline mutation and KRAS (G12R) somatic mutation. Unfortunately, the patient's condition worsened suddenly by a severe gastrointestinal hemorrhage leading to death in July 2020.

## Discussion

Pancreatic cancer is a lethal tumor with limited treatment regimens. The adoption of molecular-guided precision medicine with targeted therapy has revealed immense value in improving the treatment effect and prognosis of patients with pancreatic cancer (15, 16). We present a successful case of targeted therapy in the treatment of locally advanced pancreatic cancer after multiple-line therapies. PALB2 (D1168Efs^*^22) germline mutation and KRAS (G12R) somatic mutation were detected in the patient through NGS.

### PALB2 Mutation

PALB2 encodes a chaperone and localization protein of BRCA2 which stabilizes BRCA2 localization and promotes BRCA2 accumulation in the nucleus. PALB2 preferentially recognizes the D-loop structure of DNA, directly interacts with the recombinant RAD51, and activates the DNA insertion which is crucial for the homologous recombination repair process (17).

PALB2 has been identified as one of the major known susceptibility genes for pancreatic cancer (9). The germline mutation of PALB2, a significant gene in regulating homologous recombination and mediating DNA damage response, could cause DNA replication and damage response defects (18). In a study analyzing data from 524 families with germline PALB2 pathogenic variants, an increased risk of pancreatic cancer was observed in PALB2 pathogenic variants carriers (19). Another study has verified that the germline PALB2 mutation was associated with an increased risk of developing pancreatic ductal adenocarcinoma (PDAC) in patients with intraductal papillary mucinous neoplasms (20).

The NCCN guidelines (V1.2020) recommend that patients with metastatic or locally advanced pancreatic cancer who carry deleterious germline BRCA1/2 or PALB2 variants should be treated with platinum-containing chemotherapy for first-line treatment, and NCCN guidelines also recommend considering olaparib as maintenance treatment for patients who have a deleterious germline BRCA1/2 based on the results of the phase 3 POLO trial (21). In a single-center retrospective study, 11 patients with metastatic pancreatic cancer (6 patients with BRCA2 mutations, 2 patients with ATM mutations, 1 patient with BRCA1 mutations, 1 patient with PALB2 mutations, and 1 patient with C11orf30 mutations) who received at least first-line system therapy were treated with olaparib, and the median duration of olaparib treatment was 5 months, ORR was 18%, and the average OS was 12.35 months (22). Preliminary results of a phase II clinical trial (NCT01585805) examining a PARP inhibitor veliparib in the treatment of previously treated pancreatic cancer (carrying BRCA1/2 or PALB2 variants) showed that veliparib was well-tolerated and 4 (25%) patients who received veliparib monotherapy achieved SD for more than 4 months (23).

Our patient received Olaparib combined with irinotecan when the tumor progressed after multiple-line therapies. The patient responses well to this combined therapy; stable disease was sustained from October 2018 to November 2019.

### KRAS Mutation

RAS are common oncogenes in human tumors. Genes such as NRAS, KRAS, and HRAS are frequently mutated. KRAS mutations mainly occur in amino acid codons 12, 13, and 61, inhibiting the GTP-mediated hydrolysis process, leading to the continuous activation of RAS, and thereby promoting the malignant transformation of cells (24).

KRAS mutation is the initiating genetic event for PDAC. KRAS mutations are identified in 85% of American pancreatic cancer patients, and the vast majority of mutations occur at codon 12. Double KRAS mutations often coexist in the same pancreatic cancer lesion, which may further promote tumorigenesis (25). It is generally believed that KRAS gene mutation is a poor prognostic factor for pancreatic cancer (24).

There is no FDA approved anti-tumor drug targeting KRAS gene for now, and MEK inhibitors have shown certain clinical effects on the treatment of cancers with KRAS mutation. In a phase II clinical trial (NCT01222689) enrolled 46 patients with advanced pancreatic cancer, the patients received a combination treatment of Selumetinib (MEK inhibitor) and erlotinib (EGFR TKI), and 19 patients (41%) had stable disease, of which 12 patients kept stable for more than 12 weeks, and 12 patients showed lesion shrinkage, with a median progression-free survival of 1.9 months and a median overall survival of 7.3 months (26). In a phase I clinical trial (NCT00687622), 22 patients with KRAS mutation-positive non-small cell lung cancer were treated with trametinib, of which 8 patients showed tumor shrinkage (6–52%); 2 non-small cell lung cancer patients (1 case of KRAS mutation, 1 case of KRAS/BRAF double mutation) and 2 cases of pancreatic cancer (1 case of KRAS mutation, 1 case of unknown) showed partial remission (27). It has been verified that inhibition of KRAS → RAF → MEK → ERK signaling elicits autophagy, a process of cellular recycling that protects PDA cells from the cytotoxic effects of KRAS pathway inhibition. Hence, MEK inhibitors in combination with hydroxychloroquine, a 4-aminoquinolone which shows effects on inhibiting autophagy, may show better cancer suppressing effects. In a 68-year-old patient with metastatic pancreatic cancer, after receiving trametinib plus hydroxychloroquine treatment, the tumor marker level was reduced by 50% and the lesion was significantly reduced (28).

This patient received combination therapy of trametinib and hydroxychloroquine since January 2020. The MRI (Figure 3) indicated SD in April 2020 and a significant decrease in the CA19-9 level (Figure 1) was observed in May 2020.

Recently, an increasing number of predictive signatures/genes were identified by molecular studies for a given therapy. In early-stage PDAC patients, SMAD4 aberrations were identified as a negative prognostic marker in the gemcitabine arm, and SMAD4 alteration status was associated with erlotinib effectiveness (29). The genome-wide RNA-based gemcitabine sensitivity stratification could predict the clinical benefit of adjuvant gemcitabine in PDAC patients (30). These studies demonstrated the great importance of NGS-based sequencing technology in guiding precision therapy in PDAC. Unfortunately, the patient died in July 2020 because of a severe gastrointestinal hemorrhage. The overall survival of this locally advanced pancreatic cancer patient was ~45 months, with a progression-free survival of ~18 months for NGS-based targeted therapy after multiple-line therapies.

## Conclusion

We report a locally advanced pancreatic cancer patient who harbors PALB2 germline mutation and KRAS somatic mutation receiving targeted therapy. Fairly satisfactory efficacy of NGS-targeted therapy after multiple-line therapies was observed in this patient. This case demonstrated that targeted therapy based on the potential therapeutic targets detected by the NGS may be an important approach to improve the survival of pancreatic cancer patients.

## Data Availability Statement

The original contributions presented in the study are included in the article/Supplementary Material, further inquiries can be directed to the corresponding author/s.

## Ethics Statement

Written informed consent was obtained from the individual(s) for the publication of any potentially identifiable images or data included in this article.

## Author Contributions

WW, YL, LL, YG, WF, PZ, and AZ collected the clinical information, diagnostic information, therapeutic information, and images of the patients. MX and QH provided the NGS result and some advisable information for the result. WW wrote and submitted the manuscript. YL, YJ, LL, DL, PZ, and AZ revised the manuscript. All authors contributed to the article and approved the submitted version.

## Funding

This study was sponsored by National Natural Science Foundation of China (82074208 and 81472346), Zhejiang Provincial Natural Science Foundation (LY20H160033) and A Project Supported by Scientific Research Fund of Zhejiang Provincial Education Department (Y202045581).

## Conflict of Interest

MX and QH were employed by the company OrigiMed. The remaining authors declare that the research was conducted in the absence of any commercial or financial relationships that could be construed as a potential conflict of interest.

## Publisher's Note

All claims expressed in this article are solely those of the authors and do not necessarily represent those of their affiliated organizations, or those of the publisher, the editors and the reviewers. Any product that may be evaluated in this article, or claim that may be made by its manufacturer, is not guaranteed or endorsed by the publisher.
