# Supplementary material for: Case Report: Effectiveness of Targeted Treatment in a Patient With Pancreatic Cancer Harboring PALB2 Germline Mutation and KRAS Somatic Mutation
Source: Front Med (Lausanne). 2022 Jan 13;8:746637. doi: 10.3389/fmed.2021.746637 (PMC8792848; doi:10.3389/fmed.2021.746637)
Supplement: Supplementary file 1 [file Data_Sheet_1.pdf]

raw data-Figure 1

Time	CA199	Events
20171121	137.3	肝转移
20171212	295.3	白蛋白紫杉醇+卡培他滨化疗前
20180503	111.4	白蛋白紫杉醇+卡培他滨化疗后
20180911	1103.5	腹壁、后腹膜转移，PD。伊立替康单药治疗前
20181008	1272.6	加奥加帕利前
20190510	55.4	伊立替康+奥拉帕尼后
20191102	975.2	PD, SOX化疗前
20200111	1626.1	SOX化疗后
20200208	3852	曲美替尼+羟氯喹治疗前
20200515	1421.1	曲美替尼+羟氯喹治疗后

raw data-Figure 2

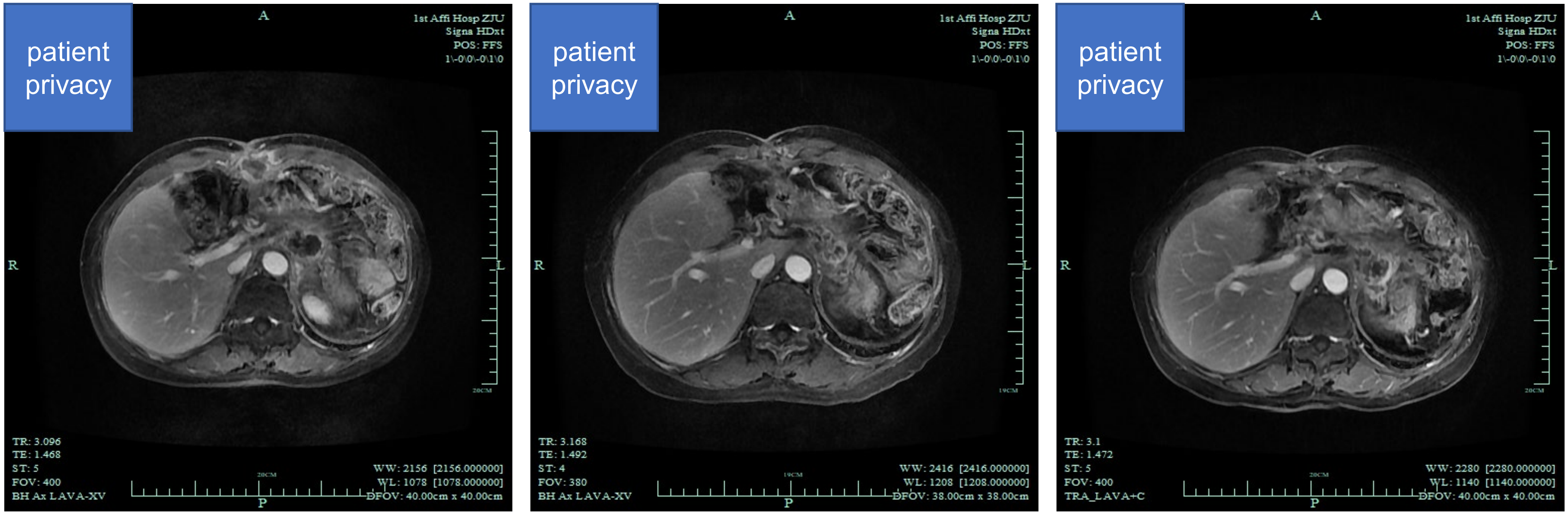

raw data-Figure 3

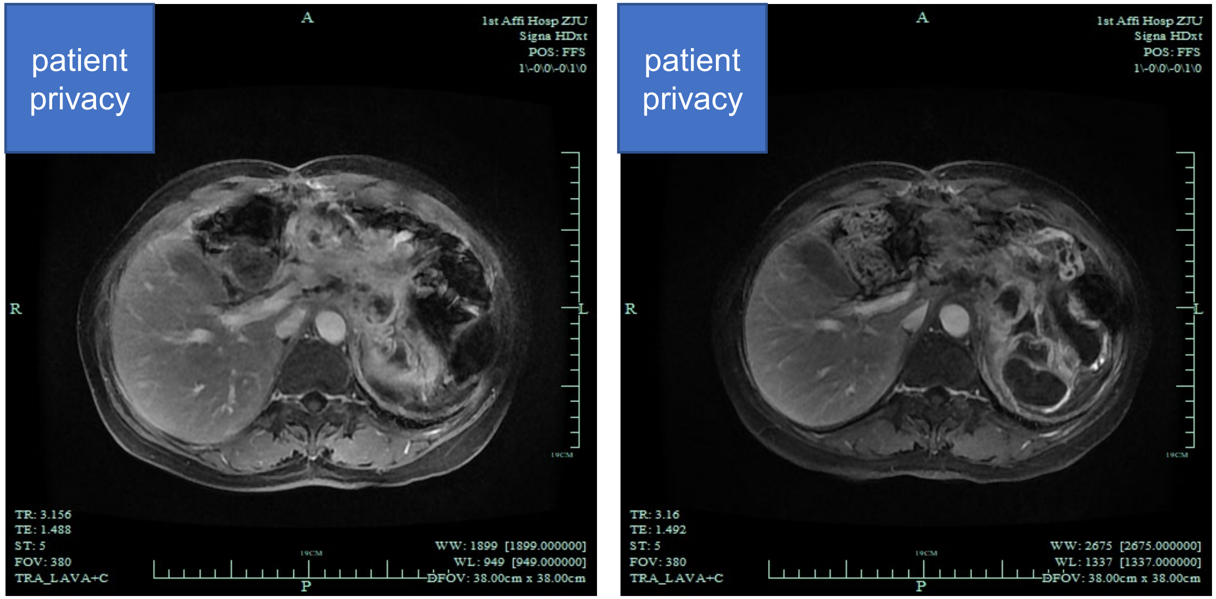

3. 检测结果汇总

3.1 基因变异检测结果汇总 450 基因检测出 4 个基因的 4 个具有临床意义的基因变异

基因名称	变异信息		可能的临床获益药物	临床实例
PALB2（胚系变异）	D1168Efs*22	exon13	临床试验	有
KRAS	G12R	exon2	临床试验	有
AXIN1	A522Rfs*183	exon6	暂无	暂无
AXIN2	S244Rfs*12	exon2	暂无	暂无
*临床实例是指针对所检测到的基因（不考虑变异形式）的临床治疗并且肿瘤缩小 30%以上的实际案例				

3.2 肿瘤突变负荷（Tumor Mutation Burden，TMB）计算结果

检测内容	计算结果	可能的临床获益药物
TMB	3.2 Muts/Mb	暂无

3.3 微卫星不稳定性计算结果

检测内容	计算结果	可能的临床获益药物
微卫星不稳定性	微卫星稳定（MSS）	暂无
